# Metabolic Reprogramming in Toll-like Receptor-Mediated Platelet Activation

**DOI:** 10.3390/cells14120906

**Published:** 2025-06-16

**Authors:** Lih T. Cheah, Jawad S. Khalil, Mary McKay, Mohammad Ali, Cedric Duval, Amanda J. Unsworth, Khalid M. Naseem

**Affiliations:** Discovery and Translational Science Department, Leeds Institute of Cardiovascular & Metabolic Medicine, University of Leeds, Leeds LS2 9JT, UK; l.t.cheah@leeds.ac.uk (L.T.C.); j.s.khalil@leeds.ac.uk (J.S.K.); m.f.ashworth@leeds.ac.uk (M.M.); ummali@leeds.ac.uk (M.A.); c.duval@leeds.ac.uk (C.D.); a.j.unsworth@leeds.ac.uk (A.J.U.)

**Keywords:** metabolic reprogramming, platelet activation, toll-like receptor

## Abstract

Beyond haemostasis and thrombosis, platelets are increasingly recognized for playing a crucial role in modulating immunoinflammation. Toll-like receptors (TLRs) constitute the first line of defence against infection and injury, with their engagement stimulating thrombotic and immune responses in platelets. Hence, anti-platelet drugs have been used to treat patients with infections and inflammation. However, due to the increased risk of bleeding with current anti-platelet drugs, alternative therapeutic targets need to be identified to ameliorate the consequences of inflammation-driven platelet hyperactivation. Previously, we demonstrated that resting platelets exhibit a metabolic plasticity that facilitates fuel selection flexibility, while in contrast, thrombin-stimulated platelets become highly glycolytic. Since multiple aspects of platelet activation require energy in terms of ATP, we investigated metabolic alterations in TLR1/TLR2-activated platelets. In this study, we have demonstrated that TLR1/TLR2-induced platelet activation reprogrammed platelets to upregulate glycolysis via CD36-linked mechanisms. In addition, we showed that this glycolytic flux is controlled by hexokinase (HK), which plays a crucial role in TLR1/TLR2-induced platelet aggregation. Targeting platelet metabolism plasticity may offer a novel strategy to inhibit platelet function in TLR-initiated diseases.

## 1. Introduction

Though primarily involved in haemostasis and thrombosis, platelets are increasingly recognized for playing a crucial role in orchestrating and sustaining the immunoinflammatory response [1]. Platelets can drive thrombo-inflammation via platelet–leucocyte interaction in infectious inflammatory conditions, including sepsis and infection [2], as well as sterile inflammatory cardiovascular diseases [3]. *In vitro* and *in vivo* studies have demonstrated that during infection, platelets and bacteria physically interact, leading to platelet activation and aggregation [4]. A family of pattern recognition receptors, known as toll-like receptors (TLRs) constitutes the first line of defence against infection and injury, recognising both pathogen-specific, pathogen-associated molecular patterns (PAMPs) and damage-associated molecular patterns (DAMPs). To date, ten members of the TLR family have been reported in platelets, including TLR 1, 2, 4, 5, 6, and 10, which are expressed on the platelet surface, as well as intracellular TLR 3, 7, 8, and 9 [5]. The presence of these TLRs in platelets highlights their prevalent innate immune sensor role within the bloodstream. The role of platelet TLRs has been extensively studied [5]. Platelet TLR1/TLR2 engagement stimulates a range of thrombotic fibrinogen [6] and PAC1 binding [7,8], aggregation [6,9], and ATP [6,10] and ADP [9] release and immune (platelet–leucocyte aggregation [6,7] and inflammatory mediator release [7,11]) responses. In addition, CD36 is a highly abundant class B scavenger receptor expressed on the surface of platelets, with about 16,700 copy numbers per human platelet [12], recognising PAMPs, DAMPs, and lipoprotein-associated molecular patterns [13]. We have previously reported that CD36 is capable of transducing plasma lipid stress into platelet hyperactivity and thrombosis through the binding of oxidised low-density lipoproteins [14,15]. Biswas *et al.* have demonstrated that cooperation of TLR2, TLR6, and CD36 is required for the hyperactivation of platelets induced by oxidised phospholipids [16], and CD36 has been shown to promote sterile inflammation via complex formation with TLR2/TLR6 [17] and TLR4/TLR6 heterodimers [18]. Anti-platelet drugs are being used to treat sepsis patients to ameliorate platelet function; however, due to the increased bleeding risk associated with these drugs, they are discontinued in critically ill patients [19]. In addition, it is reported that despite treatment with anti-platelet agents, TLR-mediated platelet activation can continue to occur [8]. Therefore, the identification of new therapeutic targets is urgently required to inhibit pathogen-stimulated platelet activation.

We and others have reported that the activation of platelets involves dramatic increases in ATP demand [20,21,22,23]. Upon activation, in common with other myeloid cells, platelets adopt a highly glycolytic phenotype regardless of nutrient availability, a process termed metabolic reprogramming [24], a phenotype analogous to the Warburg effect seen in cancer cells [25]. The aim of this study is to explore the possibility of targeting platelet metabolism as a new therapeutic intervention to prevent TLR-mediated platelet activation. We hypothesised that TLR-induced platelet activation causes platelets to undergo glycolytic metabolism reprogramming.

## 2. Materials and Methods

### 2.1. Human Platelet Isolation

Human-washed platelets (WPs) were isolated from blood taken from healthy volunteers, and all human work was approved by the School of Medicine Research Ethics Committee (MREC 19-006 and MREC 23-001, University of Leeds). Human blood was taken from drug-free volunteers by venepuncture using acid citrate dextrose (ACD; 29.9 mM sodium citrate, 113.8 mM glucose, 72.6 mM NaCl and 2.9 mM citric acid, pH 6.4) as an anticoagulant. Platelet-rich plasma (PRP) was obtained by centrifugation of whole blood at 100× *g* at 20 °C for 20 min. PRP was treated with 200 nM PGI_2_ (Sigma, Dorset, UK) and centrifuged at 1000× *g* for 10 min. The platelet pellet was then suspended in 90% (*v*/*v*) Modified Tyrode’s buffer (150 mM NaCl, 5 mM HEPES, 0.55 mM NaH_2_PO_4_, 7 mM NaHCO_3_, 2.7 mM KCl, 0.5 mM MgCl_2_, 5.6 mM glucose, pH 7.4) and 10% (*v*/*v*) ACD and then spun once more at 1000× *g* for 10 min in the presence of 200 nM PGI_2_. Platelets were finally re-suspended in Modified Tyrode’s buffer and counted using a Beckman Coulter Counter Z1 and adjusted to the indicated concentration.

### 2.2. Murine Platelet Isolation

All animal husbandry, housing, and procedures were carried out in line with the regulations and guidelines of the University of Leeds Central Biological Services Facility under the Animals (Scientific Procedures) Act 1986 and carried out under the United Kingdom Home Office approved project licence (PP0499799). Animals received standard rat and mouse no. 1 maintenance diet (RM1, Special Diet Services, Witham, UK) and water from Hydropac pouches. All mice were housed in individually ventilated cages (GM500, Techniplast, Buguggiate, Italy) with 12 h light/dark cycles at 21 °C and 50–70% humidity. Whole-body CD36 knockout (denoted as CD36KO) (Jackson Laboratory, Bar Harbour, ME, USA) wild-type (denoted as WT) mice were all on C57BL/6 backgrounds.

Murine blood was obtained by the inferior vena cava and was drawn into 1 mL syringes with 200 µL of ACD. Whole blood was diluted to a final volume of 2 mL with Modified Tyrode’s buffer. Diluted whole blood was centrifuged at 100× *g* for 5 min to isolate platelet-rich plasma (PRP), blood was diluted further to a final volume of 1 mL, and then it was centrifuged again at 100× *g* for 5 min. The final PRP was removed and pooled, then centrifuged at 1000× *g* for 6 min. The platelet pellet was then resuspended in Modified Tyrode’s buffer and adjusted to 2.5 × 10^8^ platelets/mL.

### 2.3. Light Transmission Platelet Aggregation

WPs (2.5 × 10^8^ platelets/mL) were incubated with inhibitors or Modified Tyrode’s buffer at the indicated concentration at 37 °C for 15 min before aggregation with the addition of 20 µg/mL Pam3CSK4 (Invitrogen, Carlsbad, CA, USA), and it was monitored under constant stirring (600 rpm) for 6 min using Helena AggRAM (Helena Biosciences Europe, Gateshead, UK). To examine the effect of HK and CD36 on Pam3CSK4-induced platelet aggregation, WPs were pre-treated with vehicle, 2-deoxyglucose (50 mM 2DG, Sigma), 3-bromopyruvate (30 µM 3BP, Stratech, Ely, UK), or sulfo-N-succinimidyl oleate (1 µM SSO, Santa Cruz, Dallas, TX, USA) for 15 min before the addition of Pam3CSK4.

### 2.4. Bioenergetic Profiles

The Agilent Seahorse XFe96 Analyser (Agilent Technologies, Cheshire, UK) was used to measure the platelet extracellular acidification rate (ECAR, a measure of glycolysis) and oxygen consumption rate (OCR, a measure of oxidative phosphorylation). The XF cell culture microplate was coated with 22.4 µg/mL Cell-Tak adhesive [26] (Corning Inc., Corning, NY, USA) for 1 h at 37 °C and was washed with PBS twice prior to experiments. WPs (2 × 10^8^ platelets/mL, 50 µL) resuspended in Seahorse XF DMEM medium (pH 7.4, Agilent Seahorse Bioscience, Cheshire, UK) supplemented with 5 mM glucose, 1 mM pyruvate, and 2 mM glutamine were seeded onto each well and subjected to 1 min, 100× *g* centrifugation in two opposite directions. The volume of each well was adjusted to 180 µL with DMEM. Platelets were stimulated with vehicle, 0.05 U/mL thrombin (Sigma), or 20 µg/mL Pam3CSK4 in the Agilent Seahorse real-time ATP rate assays, which were carried out according to the manufacturers’ instructions (Agilent Seahorse Bioscience, Cheshire, UK, 103592-100). In brief, the electron transport chain inhibitors 1.5 µM oligomycin and 0.5 µM rotenone/antimycin A were injected sequentially to allow for the calculation of oxidative phosphorylation, as well as glycolysis-mediated ATP production rates (mito, glyco) from resulting OCR and ECAR. MitoATP and glycoATP production rates were obtained from the Agilent Seahorse real-time ATP rate assay report generator.

### 2.5. Hexokinase Activity Assay

WPs (5 × 10^8^ platelets/mL) were pre-treated with vehicle, 2DG, or 3BP for 15 min followed by 20 µg/mL Pam3CSK4 treatment for 30 min. Then, an equal volume of CelLyticTM MT cell lysis reagent (Sigma) was added to the platelet suspension and was rested on ice for 30 min before centrifugation at 14,000× *g* for 2 min at 4 °C in the presence of protease and phosphatase inhibitors. Platelet cytosolic fractions were aliquoted for HK activity, a colorimetric assay (Abcam, Cambridge, UK, ab136957) which was carried out according to the manufacturers’ instructions.

### 2.6. Statistical Analyses

Results are expressed as means ± SD, and statistical analyses were undertaken using Prism 9.5 (Graphpad, Boston, MA, USA). Comparisons between groups were performed by an unpaired *t*-test with Welch’s correction, a one-way ANOVA, or a two-way ANOVA with post hoc Tukey’s test correction. *p* values of less than 0.05 were considered statistically significant.

## 3. Results and Discussion

### 3.1. TLR1/TLR2 Engagement Induces Platelet Aggregation via CD36-Mediated Mechanism

The dimerization of TLR2 with its co-receptor TLR1 enables it to identify a range of pathogens. Previous studies have demonstrated that synthetic triacylated lipopeptide Pam3CSK4, which activates the innate immune system through TLR1/TLR2 heterodimers [27,28], induces platelet aggregation, α- and dense granule secretion, and platelet–neutrophil aggregate formation [6]. In contrast, lipopolysaccharide (LPS), a TLR2/TLR4 agonist, does not elicit classical platelet activation [29]. The mechanism underlying TLR1/TLR2-induced platelet activation remains unclear. CD36 promotes inflammation via TLR2/TLR6 [17] and TLR4/TLR6 [18]; we therefore tested the role of CD36 in TLR1/TLR2-activated platelet aggregation.

**Figure 1:**
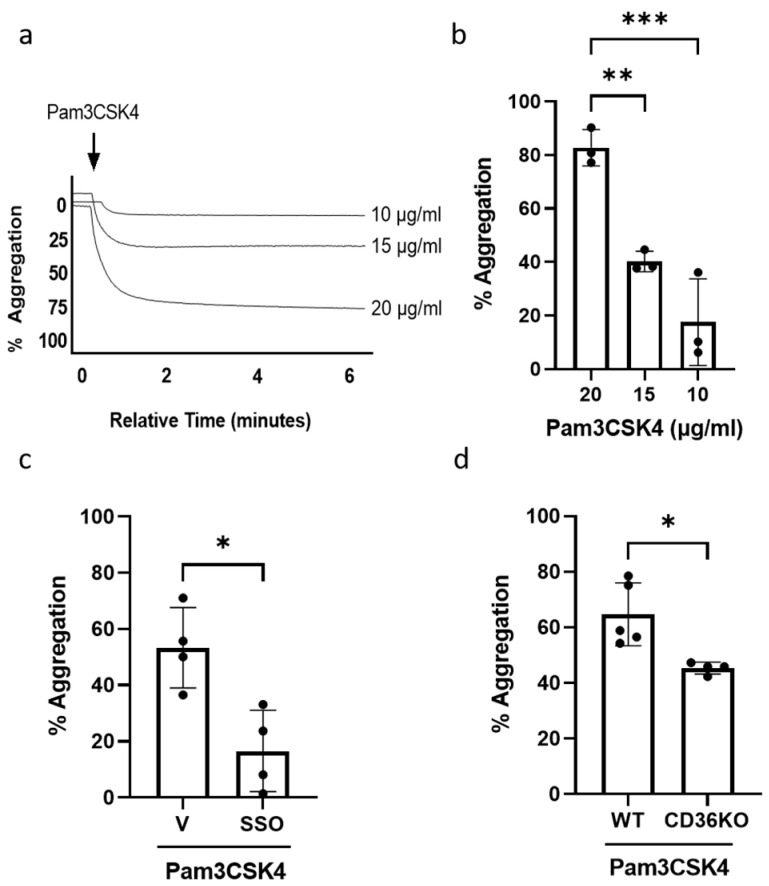
TLR1/TLR2-stimulated platelet aggregation occurs through a CD36-mediated mechanism. WPs (2.5 × 10^8^ platelets/mL) from healthy volunteers were stimulated with either 10, 15, or 20 µg/mL of Pam3CSK4 and aggregation was recorded for 6 min. Representative aggregation traces (**a**); the percentage of aggregation (**b**), expressed as mean ± SD (n = 3). WPs (2.5 × 10^8^ platelets/mL) from healthy volunteers were pre-treated with vehicle (V) or 1 µM SSO for 15 min before the addition of 20 µg/mL Pam3CSK4 in the aggregation experiment (**c**). WPs (2.5 × 10^8^ platelets/mL) isolated from WT and CD36KO were stimulated with 20 µg/mL Pam3CSK4 in the aggregation experiment (**d**). Data are expressed as mean percentage of aggregation ± SD (n = 4–5). Data were analysed by a one-way ANOVA followed by a post hoc test (Tukey’s) or unpaired *t*-test: * *p* < 0.05, ** *p* < 0.01, *** *p* < 0.001.

We first confirmed that Pam3CSK4 induces platelet aggregation in human platelets and observed a dose-dependent increase in aggregation following the treatment of platelets with increasing concentrations of Pam3CSK4 (Figure 1a,b). SSO, a CD36-blocking agent, has been shown to inhibit CD36 by irreversibly binding to the lysine 164 [30]. When SSO was used to pre-treat the human platelets before Pam3CSK4 stimulation, platelet aggregation was significantly inhibited (Figure 1c). In CD36KO murine platelets, Pam3CSK4-induced aggregation was inhibited compared to the WT group (Figure 1d). These data have demonstrated that platelet aggregation initiated by TLR1/TLR2 activation occurs via the CD36-mediated pathway in both human and murine platelets.

### 3.2. TLR1/TLR2-Mediated Platelet Activation Is Glycolytically Driven

**Figure 2:**
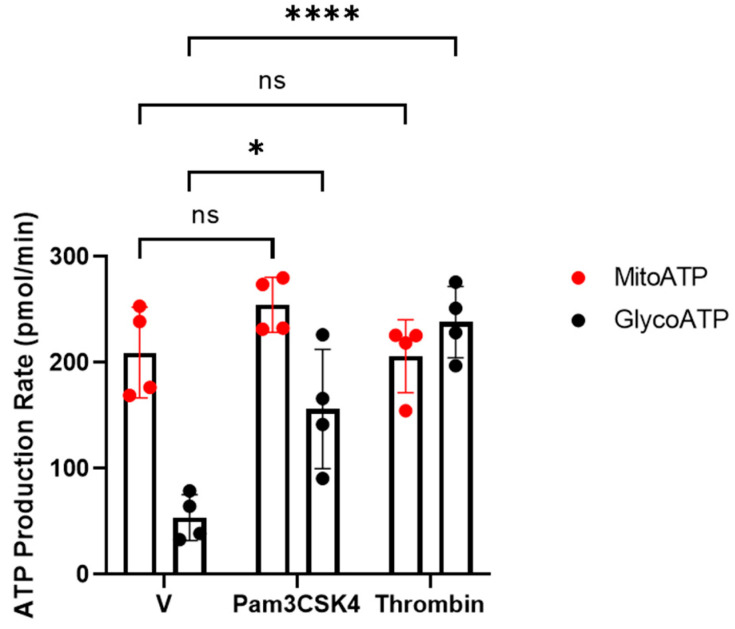
Changes in ATP production rates in response to Pam3CSK4 and thrombin. WPs (2 × 10^8^ platelets/mL) from healthy volunteers were stimulated with vehicle (V), 0.05 U/mL thrombin, or 20 µg/mL Pam3CSK4 and the mito- (red) and glyco-ATP (black) production rates were measured using the Agilent Seahorse real-time ATP rate assay. Data are expressed as mean ± SD (n = 4). Data were analysed by a two-way ANOVA followed by a post hoc test (Tukey’s): * *p* < 0.05, **** *p* < 0.0001.

Previously, we and others have reported that the activation of platelets requires dramatic increases in the demand of ATP [20,21,22,23]. Therefore, we examined the contribution of ATP production rates from glycolysis and mitochondrial oxidative phosphorylation on TLR1/TLR2-mediated platelet activation. To address this, an ATP rate assay was measured by using the Seahorse bioenergetic analyser. Under basal conditions, there is approximately 80% of mitoATP and 20% of glycoATP, indicating that platelets predominantly rely on ATP produced from mitochondria in the quiescence state (Figure 2). When platelets were activated by physiological haemostasis agonist thrombin, glycoATP was significantly upregulated, whereas mitoATP remain unaffected. However, when platelets were challenged by Pam3CSK4, ATP produced from glycolysis was significantly enhanced and those from mitochondria appeared to slightly increase but did not reach significance. This indicates that TLR1/TLR2-mediated platelet activation is mainly glycolytically driven. Interestingly, Claushuis *et al.* (2019) have reported that the stimulation of platelets with LPS, a TLR2/TLR4 agonist, did not cause classical platelet activation but induced an increase in mitochondrial maximal respiration [29]. The inhibition of TLR4 prior to LPS stimulation abolished the increase in maximal respiration, which was not observed when TLR2 was inhibited, indicating that the mitochondrial effect in response to LPS is primarily mediated via TLR4. Taken together, these observations suggest that the activation of specific TLR isoforms regulates different metabolic pathways in platelets.

### 3.3. Inhibition of HK Prevents TLR1/TLR2-Mediated Platelet Aggregation

**Figure 3:**
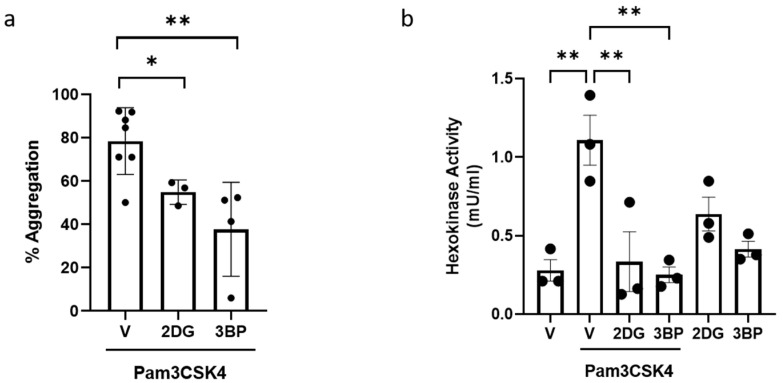
TLR1/TLR2-induced platelet aggregation is HK-dependent. WPs (2.5 × 10^8^ platelets/mL) from healthy volunteers were pre-treated with vehicle (V), 50 mM 2DG, or 30 µM 3BP for 15 min before the addition of 20 µg/mL Pam3CSK4 in the aggregation experiment (**a**). Results are expressed as mean percentage of aggregation ± SD (n = 3–7). WPs (5 × 10^8^ platelets/mL) were pre-treated with vehicle (V), 50 mM 2DG, or 30 µM 3BP for 15 min prior to stimulation with or without 20 µg/mL Pam3CSK4 for 30 min (**b**). HK activity was determined. Data are expressed as mean ± SD (n = 3). Data were analysed by a one-way ANOVA followed by a post hoc test (Tukey’s): * *p* < 0.05, ** *p* < 0.01.

As Pam3CSK4 activation increased the level of glycoATP in platelets, we then evaluated whether this increase in glycolysis was essential for Pam3CSK-mediated platelet activation or whether the level of mitoATP was sufficient to meet cellular ATP requirements. Hexokinase (HK) catalyses the first step in glucose metabolism in the cells, converting glucose to glucose-6 phosphate. HKs exist as three isoforms (HK 1–3) and the related glucokinase [31]. Proteomic and biochemical studies indicate that HKs are expressed in platelets [12,32]. When platelets were pre-treated with a pan-HK inhibitor, 2DG [33,34], Pam3CSK4-induced aggregation was reduced to 70% (Figure 3a). Moreover, greater inhibition (approximately 50%) was observed following pretreatment with HK2-specific inhibitor 3BP [35]. In further support of the TLR1/TLR2-dependent regulation of glycolysis, Pam3CSK4 was found to significantly upregulate HK activity in platelets, which was abolished in the presence of both 2DG and 3BP (Figure 3b). Taken together, these data demonstrated that TLR1/TLR2-induced platelet aggregation and activation require metabolic reprogramming and an increase in platelet glycolysis that is mediated by HK activation.

## 4. Conclusions

We have demonstrated for the first time that TLR1/TLR2-induced platelet activation rewires platelets to upregulate glycolysis to support platelet activation via CD36-mediated mechanisms. In addition, we showed that HK, a key enzymatic step controlling glycolytic flux, plays a critical role in TLR1/TLR2-induced platelet aggregation. In conclusion, targeting platelet metabolism plasticity may be explored as a novel strategy to inhibit TLR-initiated platelet function.

## Data Availability

The original contributions presented in this study are included in the article. Further inquiries can be directed to the corresponding author.
